# Competing Bioaerosols May Influence the Seasonality of Influenza-Like Illnesses, including COVID-19. The Chicago Experience

**DOI:** 10.3390/pathogens10091204

**Published:** 2021-09-16

**Authors:** Richa B. Shah, Rachna D. Shah, Damien G. Retzinger, Andrew C. Retzinger, Deborah A. Retzinger, Gregory S. Retzinger

**Affiliations:** 1 Department of Psychology, Northwestern University, Evanston, IL 60209, USA; richashah2023@u.northwestern.edu; 2 Department of Medicine, Stritch School of Medicine, Loyola University, Chicago, IL 60153, USA; drshah@allergydrshah.com; 3 Department of Computer Science, Graycore, Cincinnati, OH 45140, USA; damien@graycore.io; 4 Department of Emergency Medicine, West Virginia University, Camden Clark Medical Center, Parkersburg, WV 26101, USA; andrew.retzinger@gmail.com; 5 Independent Researcher, Cincinnati, OH 45140, USA; zingsdar@gmail.com; 6 Department of Pathology, Feinberg School of Medicine, Northwestern University, Chicago, IL 60611, USA

**Keywords:** influenza-like illness (ILI), COVID-19, SARS-CoV-2, pollens, mold spores, bioaerosols, innate immunity, Toll-like receptor, TLR4, fibrin(ogen) D-domain

## Abstract

Data from Chicago confirm the end of flu season coincides with the beginning of pollen season. More importantly, the end of flu season also coincides with onset of seasonal aerosolization of mold spores. Overall, the data suggest bioaerosols, especially mold spores, compete with viruses for a shared receptor, with the periodicity of influenza-like illnesses, including COVID-19, a consequence of seasonal factors that influence aerosolization of competing species.

## 1. Introduction

Influenza-like illnesses (ILIs) attributable to influenza viruses and to coronaviruses are sharply seasonal [1,2]. Importantly, recent data from the Netherlands indicate there exists an inverse relationship between the seasonal incidence of ILIs, including COVID-19, and pollen count [3,4]. To discern whether such a relationship might be the case generally, pollen count in Chicago was related to ILIs reported by local emergency departments. In Chicago, as in the Netherlands, ILIs fall as total pollen count rises.

Because bioaerosols measured in Chicago include not only pollens but also mold spores, ILIs were related to counts of the two measured species. Just as they do for pollens, ILIs fall as mold spores rise. In contradistinction to their temporal relationship with pollens, however, ILIs remain low when mold spores are high, rising again when mold spores fall.

Perusal of the various measured pollens and mold spores reveals many are echinulated, having protuberances reminiscent of those of influenza and SARS-CoV-2 virions [5,6,7,8,9,10,11]. In addition, although interactions of viral ‘spikes’ with other host proteins have been documented [12,13], such protuberances seem ideally suited to interacting especially with Toll-like receptors in a fashion akin to ‘hook-and-loop’ adhesives. Indeed, such interactions are well described for a number of pollens and mold spores, and there is no reason to believe viruses, despite their smaller size, interact differently. Implicating Toll-like receptors, Toll-like receptor 4 (TLR4) in particular, seems appropriate on phenomenological grounds as well: (1) engagement of TLR 4 can account for the inflammatory signaling characteristic of severe COVID-19 [14,15], (2) TLR4 is intimately involved in the inflammation elicited both by sharply seasonal respiratory viruses and by multiple species of fungi [16,17,18,19,20], (3) COVID-19 prognosis correlates with radiographic involvement of alveoli [21,22], the epithelial cells of which are rich in TLR4 [23], (4) age-dependent hyper-responsiveness of TLR4 [24], especially in the context of TLR5 [25,26], can account for the age dependent severity of COVID-19, and (5) fibrinogen D-dimers, composites of TLR4 ligands [27], are markedly elevated in persons with severe COVID-19 [28].

In this report, inverse relationships between seasonal expression of pollens and seasonal presentations of ILIs and COVID-19 are confirmed. More importantly, it is shown that mold spores, which over a season constitute the dominant bioaerosol, also have an inverse relationship with the respiratory illnesses. As to what accounts for the inverse relationships, it appears certain bioaerosols—especially mold spores—compete with viruses for an effector of innate immunity, likely TLR4, thereby limiting viral engagement and, consequently, ILIs.

## 2. Materials and Methods

### 2.1. Collection and Counting of Pollens and Mold Spores

A volumetric spore trap (Burkard Manufacturing, Hertfordshire, England) equipped with a 24 h sampling head was used to collect pollens and mold spores. The trap was fixed ~70 feet above ground, on a roof in Melrose Park, Il, USA. A standard glass microscope slide coated with grease was placed in a carriage that moved at a rate of 2 mm/h past the trap orifice (14 mm × 2 mm). Air was drawn through the orifice at a rate of 10 L/min, thereby impacting airborne particles against the greased slide. Slides so exposed were stained with glycerin jelly supplemented with basic fuchsin. After applying a coverslip, a slide was evaluated microscopically for both pollens and mold spores, Table 1. The quantities of the various pollens and mold spores are available in Supplementary Tables S1 and S2, respectively. A new slide was placed in the trap daily and the carriage was re-oriented to its start position. Counts were made Monday through Friday, between mid-March and mid-October, the only time during which the measured bioaerosols are quantifiable.

### 2.2. ILI and COVID-19 Data

ILI data pooled from 23 large hospitals in Chicago over the period 9 January 2015 through 18 July 2020 were obtained from the Chicago Department of Public Health (CDPH). The 23 hospitals were chosen because they alone of Chicago-area hospitals consistently reported ILI presentations over the entirety of the study interval. In Chicago, the designation of ILI by emergency departments is based on fever (≥100 °F) and respiratory symptoms, i.e., cough and/or sore throat, not on any specific diagnosis. The data are included in Supplementary Table S3. COVID-19 data from all Chicago hospitals were obtained through portals of the CDPH, https://www.chicago.gov/city/en/sites/covid19/home/covid-dashboard.html (accessed on 18 July 2020) and https://data.cityofchicago.org/browse?limitTo=datasets&sortBy=alpha&tags=covid-19 (accessed on 18 November 2020). Those data are also included in Supplementary Table S4.

### 2.3. Data Analysis

Time- or dose-dependent data were paired with the corresponding time or dose and fit to equations described in the text. The best values for the parameters of the equations, as well as their corresponding 95% confidence intervals, were then determined using the paired data and a nonlinear least squares regression method [29].

## 3. Results

### 3.1. Kinetics of Presentations to Emergency Departments

**Figure 1:**
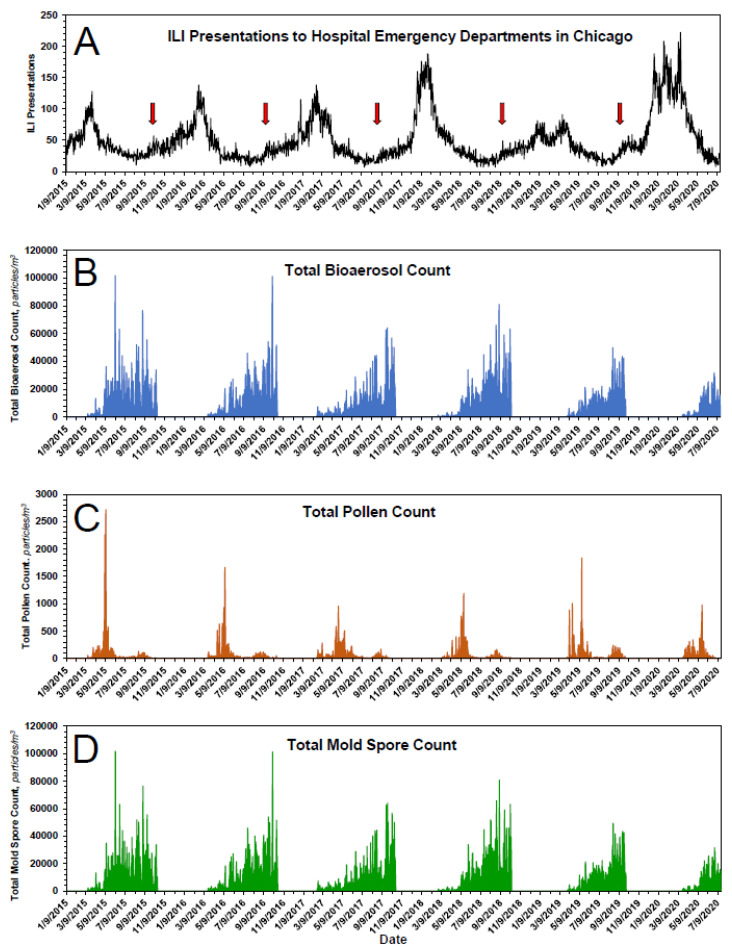
Seasonal time courses, 2015–2020, of measured variables of this study. (**A**) ILI presentations to emergency departments of hospitals in Chicago. Red arrow indicates the leading seasonal ‘bump’ in ILI presentations. (**B**) Total bioaerosol count as a function of time. (**C**) Total pollen count as a function of time. (**D**) Total mold spore count as a function of time. See text for additional details.

**Figure 2:**
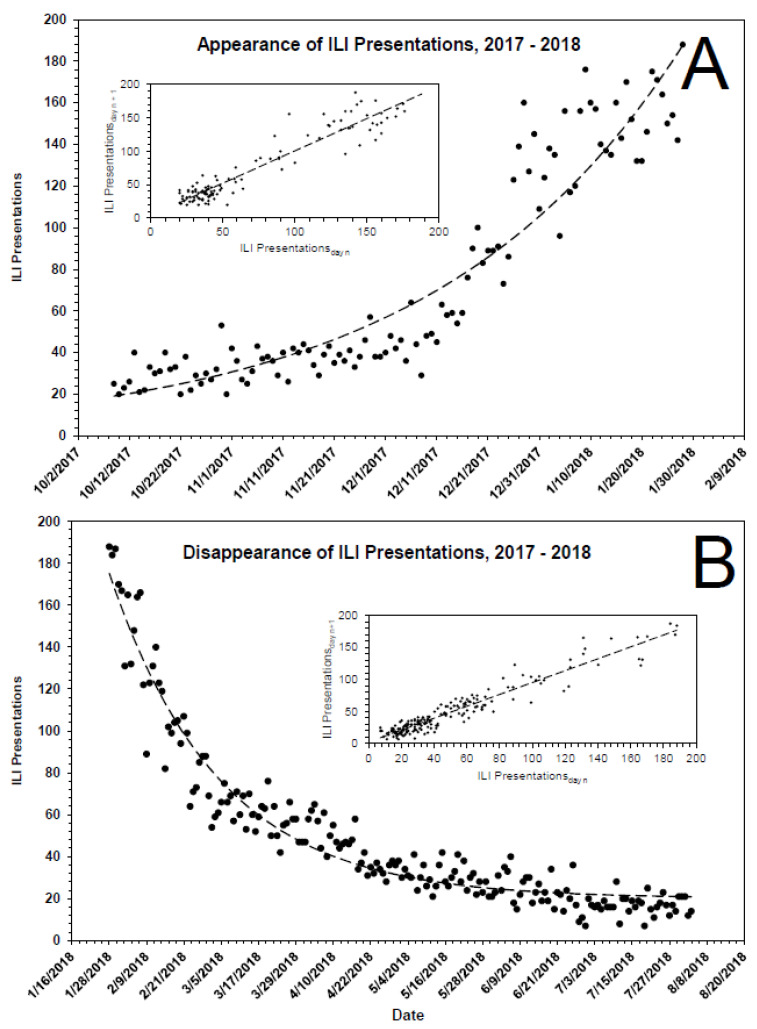
Kinetics of ILI presentations to hospitals in Chicago during a representative flu season, 2017–2018. Both the rate of appearance (**A**) and the rate of disappearance (**B**) of ILI presentations are approximately first-order. The dashed lines displayed in the primary plots are the theoretical fits of the data (Figure 1A) to a first-order rate equation, the parameters of which are given in Table 2. The inset shows the expected linearity of the same data when plotted according to the method of Kézdy [30], in this case ILI presentations_day n_ vs. ILI presentations_day n+1_.

As shown in Figure 1A, ILI presentations to emergency departments in Chicago are cyclical in nature, with a periodicity of ~1 year. The peak incidence occurs approximately during February, with the annual nadir occurring approximately during August. Over the entirety of a seasonal cycle, ILI presentations never reach zero. Although there are subtleties associated with the kinetics of ILIs for each of the individual years 2015 through 2020, the annual increase in ILI presentations for each season is characterized by a leading ‘bump,’ followed thereafter by a major rise. The relevance of the bump is addressed below, under both **Results** and **Discussion**. As for the major rise, it is approximated empirically by a first-order process, ILI_obs_ = ILI_0_e^kt^ + C, where ILI_obs_ is the number of observed ILIs during the growth phase of a cycle; ILI_0_ is the number of ILI presentations at the start of a cycle; t is time, in d; k is a first-order rate constant, in d^−1^; and C is the number of background presentations ‘masquerading’ as ILIs (Table 2 and Figure 2A). The rate of decrease from any yearly maximum also fits reasonably well a first-order process (Table 2 and Figure 2B). Taken at face value, the data suggest: (1) a not insignificant number of ILI presentations are due to pathogens other than influenza virus, e.g., parainfluenza virus, respiratory syncytial virus, measles, mumps, etc., and (2) the annual rate of change in influenza cases is due to change in ambient concentration of influenza virus.

**Figure 3:**
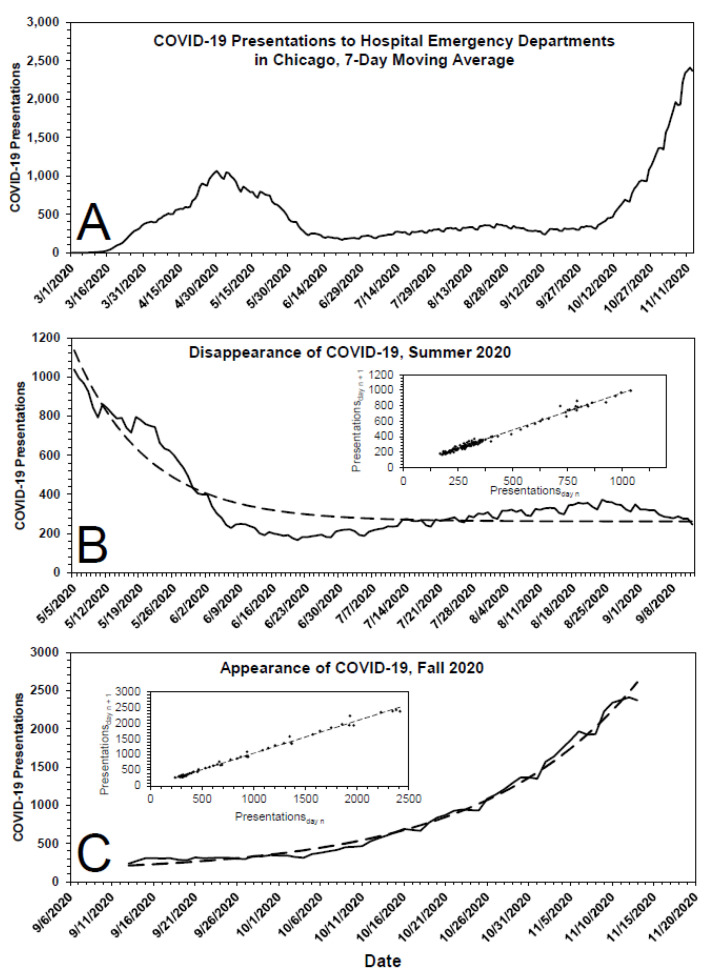
Time course and kinetics of COVID-19 presentations to all Chicago hospitals, March 2020–November 2020. (**A**) COVID-19 presentations to all hospitals in Chicago, 7-day moving average, 1 March 2020 to 14 November 2020. (**B**) Disappearance of COVID-19 presentations, 5 May 2020 to 13 September 2020. The dashed line displayed in the primary plot is the theoretical fit of the data to a first-order rate equation, the parameters of which are given in the text. For more refined analysis, see [31]. (**C**) Appearance of COVID-19 presentations, 14 September 2020 to 14 November 2020. The dashed line displayed in the primary plot is the theoretical fit of the data to a first-order rate equation, the parameters of which are given in the text. The insets show expected linearities of the data when plotted according to the method of Kézdy [30], in these cases COVID-19 presentations_day n_ vs. COVID-19 presentations_day n+1_.

Figure 3A shows the time course of the 7-day moving average of COVID-19 presentations to emergency departments of all Chicago hospitals. Because reporting was not uniformly rigorous before 1 May 2020, COVID-19 presentations prior to that date have been excluded from analyses. As shown in Figure 3B, the fall in presentations for the period 5 May 2020 through 27 September 2020 was roughly first-order, with parameters k = 0.065 d^−1^ (t_1/2_ ~ 10.6 d), COVID-19_0_ = 875 presentations and C = 262 presentations. Inasmuch as masks and physical-distancing were mandated in Chicago on 1 May 2020, the rate of fall in COVID-19 presentations, i.e., the shape of the curve, after that date was influenced to some extent by those measures, as discussed elsewhere [31].

Starting in late September 2020, COVID-19 cases in Chicago surged in first-order fashion (Figure 3C), the parameters of which are k = 0.053 d^−1^ (t_1/2_ ~ 13 d), COVID-19_0_ = 96 presentations and C = 117 presentations. Taken at face value, the data suggest: (1) some of the individuals for whom a diagnosis of COVID-19 was made did not have COVID-19 and (2) changes in the number of COVID-19 presentations in Chicago were due to changes in the ambient concentration of SARS-CoV-2.

### 3.2. Kinetics of Pollen and Mold Spore Counts

Pollens are fertilizing elements of flowering plants whilst mold spores are reproductive elements of fungi. In published studies [3,4], pollens alone were counted and related to ILIs. Left uncounted were mold spores, important seasonal contributors to the total bioaerosol burden. For the studies reported herein, both pollens and mold spores were counted. Those counts were then analyzed, in aggregate and individually.

In Chicago, pollens and mold spores are monitored from approximately mid-March to approximately mid-October, the only time during which the bioaerosols are easily measurable and also the time most problematic for persons suffering from seasonal allergies. As expected, the data indicate bioaerosol expression is cyclical with a periodicity of ~1 year (Figure 1B). The total bioaerosol count peaks during approximately mid-September and falls sharply thereafter. Data following the peaks are somewhat limited, their collection being truncated on an arbitrary end date, i.e., approximately mid-October.

**Figure 4:**
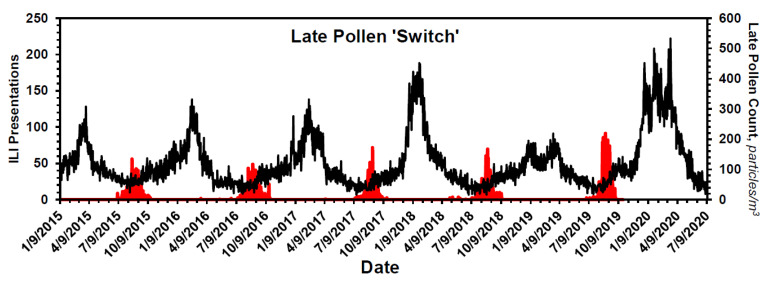
Late pollens signal the start of the flu season. Data shown in red are solely the counts of late pollens, most of which are *Ambrosia* and other *Asteraceae*. Superimposed on them are the ILI data of Figure 1A. As if a switch, the peak in the count of late pollens always occurs coincident with onset of the bump in seasonal ILI presentations. See text for additional details.

In the case of pollens, the seasonal distribution is bimodal, with a dominant first mode that peaks in approximately mid-May and a smaller second mode that peaks in approximately late August (Figure 1C). The pollens that constitute the second mode, here termed ‘late pollens’, are predominantly *Ambrosia* and the other *Asteraceae*. Importantly, the peak of the second mode always coincides with the leading bump in ILI presentations (Figure 1A and Figure 4). The potential relevance of this is addressed in the **Discussion**. In the case of mold spores, *which constitute the bulk of the measured bioaerosols* (Figure 1B,D and Table 3), the peak count, which occurs during approximately late September, falls precipitously by approximately mid-October, with an empiric half-life of ~10 d, Table 4.

**Figure 5:**
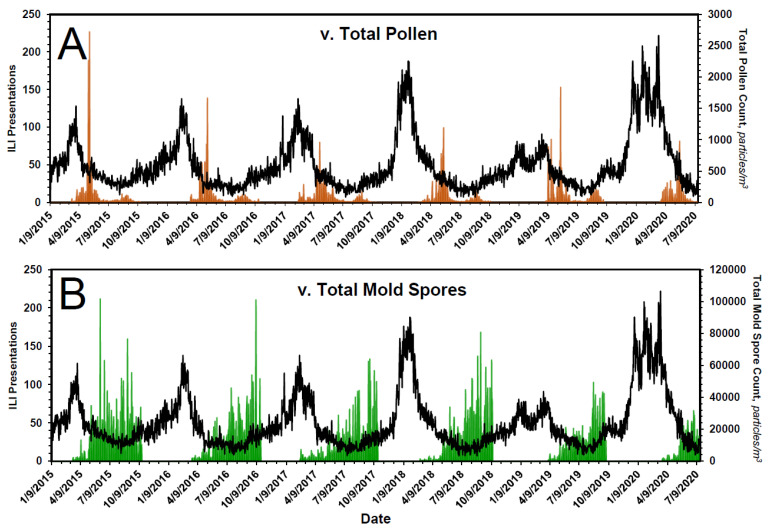
Bioaerosol expression and ILI presentations in Chicago, 2015–2020. In (**A**), the expression of pollens, in brown, is superimposed on the time course of ILI presentations. In (**B**), the expression of mold spores, in green, is superimposed on the time course of ILI presentations. The expression of pollens is bimodal, with the onset of the first mode coinciding with the drop in seasonal ILI presentations. The peak of the second mode coincides with the onset of the leading ‘bump’ in ILI presentations, the start of flu season. See text and Figure 4 for additional details. The onset of aerosolization of mold spores also coincides with the drop in seasonal ILI presentations. Thereafter, mold spore count increases across the entirety of the summer months—during which time ILIs remain low—and falls precipitously from mid-September to mid-October, at which time ILI cases begin to rise. See text for additional details.

**Figure 6:**
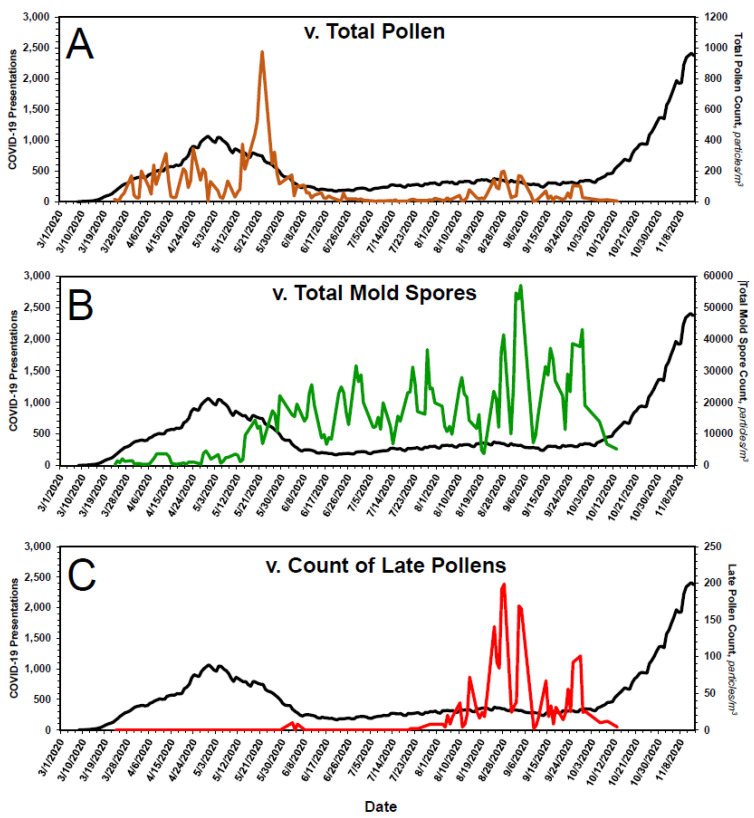
Bioaerosol expression and COVID-19 presentations in Chicago, March 2020–November 2020. In (**A**), the expression of pollens, in brown, is superimposed on the time course of COVID-19 presentations. In (**B**), the expression of mold spores, in green, is superimposed on the time course of COVID-19 presentations. In (**C**), the expression of late pollens alone, in red, is superimposed on the time course of COVID-19 presentations. As they do for ILI presentations (Figure 4), late pollens presage the onset of COVID-19 presentations in the fall. The onset of aerosolization of mold spores coincides with the drop in COVID-19 presentations in the spring. Thereafter, mold spore count increases across the entirety of the summer months—during which time COVID-19 presentations remain low—and fall precipitously from mid-September to mid-October, at which time COVID-19 presentations begin to rise. See text for additional details.

**Figure 7:**
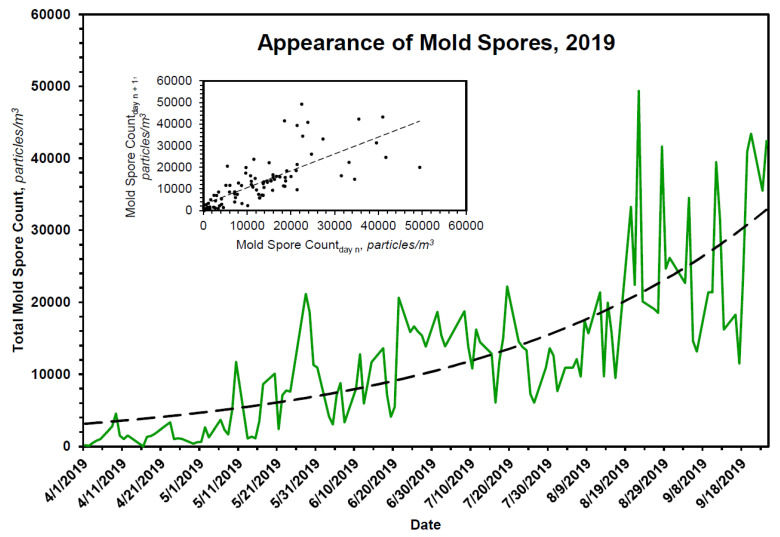
Kinetics of mold spore expression in Chicago during a representative season, 2019. The rate of appearance of mold spores in Chicago is approximately first order. The dashed line displayed in the primary plot is the theoretical fit of the data to a first-order rate equation, the parameters of which are given in Table 4. The inset shows the expected linearity of the same data when plotted according to the method of Kézdy [30], in this case mold spore count_day n_ vs. mold spore count_day n+1_.

Although these data substantiate the claim of an inverse relationship between the onset of pollen season and the end of flu season, pollen count declines rapidly and is not elevated when ILI presentations (Figure 5A) and COVID-19 presentations (Figure 6A) are low. Mold spores, on the other hand, increase continuously in first-order fashion (Table 4 and Figure 7), beginning just prior to or coincident with the fall in ILI (Figure 5B) and COVID-19 presentations (Figure 6B), and across the entirety of the summer months, when influenza and COVID-19 cases are low.

### 3.3. Inhibition of ILI and COVID-19 Presentations by Mold Spores

**Figure 8:**
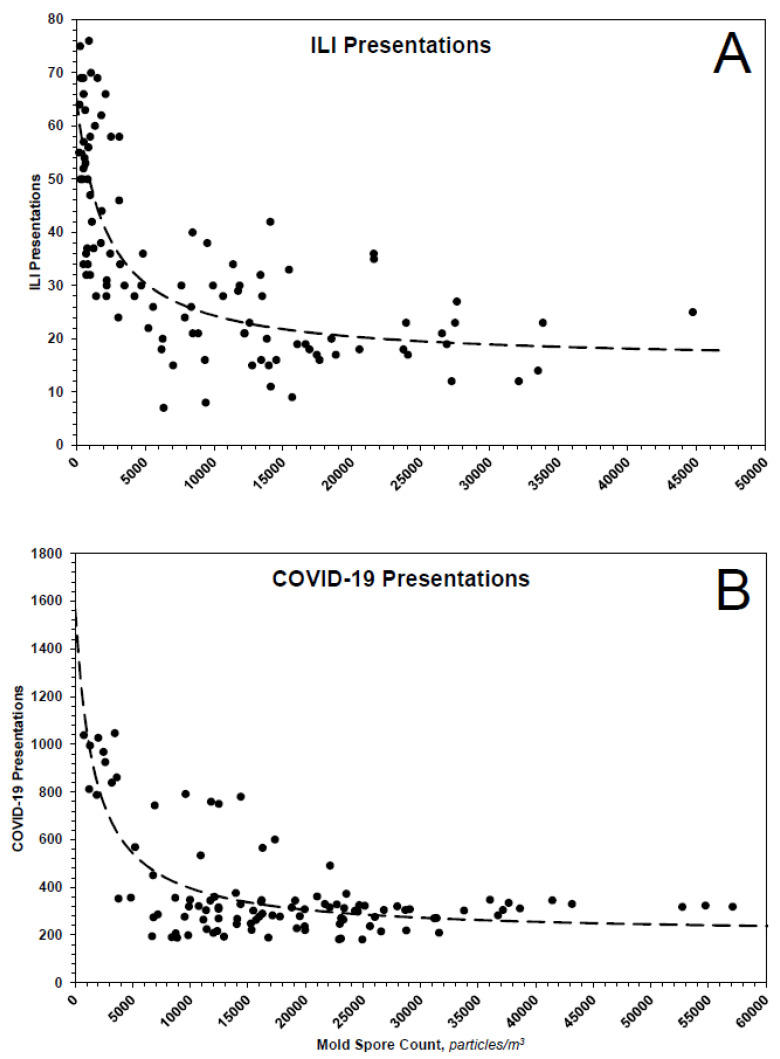
Inhibition of ILIs (**A**) and COVID-19 (**B**) by mold spores. The dotted lines represent theoretical fits of data according to the equation and parameters given in the text.

If one assumes ILI and COVID-19 presentations are consequences of the binding of relevant viruses to specific receptors, then one can treat the presentations as proxies for those receptors, for which mold spores compete. Toward that end, ILI and COVID-19 presentations were plotted as functions of total mold spore count (Figure 8). Because the curvatures of the plots suggest true equilibria, the data of each were fit to the equation P = P_o_/(1 + C/K_d_) + B, where P is the observed number of presentations to emergency departments; P_o_ is the maximum number of such presentations; C, in mold spores/m^3^, is the measured mold spore count; K_d_, in mold spores/m^3^, is the apparent dissociation constant of the receptor–mold spore complex; and B is a constant representing presentations not influenced by mold spores. As shown in the figures, the data of each plot fit the theoretical model reasonably well. From the ILI data, one calculates P_o_ ~ 50 presentations, K_d_ ~ 2128 mold spores/m^3^ and B ~ 16 presentations; from the COVID-19 data, one calculates P_o_ ~ 1366 presentations, K_d_ ~ 1668 mold spores/m^3^ and B ~ 201 presentations. The most parsimonious explanation for the near equivalence of the apparent dissociation constants is a shared receptor.

## 4. Discussion

The data presented herein are consistent with those presented earlier by others [3,4], namely, the incidence of ILIs falls as pollen count rises. Because the data of the present study derive from an urban area in North America (Chicago, IL, USA: latitude 41.85003, longitude −87.65005) whilst those of the earlier study derive from North Central Europe (Helmond, the Netherlands: latitude 51.48167, longitude 5.66111), it appears the inverse relationship may be generally valid.

Made blatantly obvious by these studies are the seasonalities of ILIs, pollens and mold spores. The annual periodicities of the three indicate the rotation of the earth about the sun is ultimately responsible. Special note should be made of: (1) the nearness of the onset of bioaerosol expression to the vernal equinox, i.e., when the lengths of day and night are nearly equal, (2) the nearness of the peak in pollen count (excepting *Ambrosia*) to the summer solstice, i.e., the longest day of the year and (3) the nearness of the peak in mold spore count to the autumnal equinox. Because light and heat from the sun are drivers of both natural and agricultural growing seasons, these dates and their relevance to the expression and dispersal of bioaerosols should come as no surprise.

With special regard to the late pollens, changes in their atmospheric concentration invariably coincide with the annual leading bump in ILIs. Inasmuch as *Ambrosia*, the dominant species, is a major respiratory allergen, the leading bump may represent ragweed sensitivities manifesting as ILI. Alternatively, it may just represent enhanced spread of respiratory viruses by, for example, school openings. Regardless, the peak in late pollen count—as if a switch—presages the major upswing in ILI (Figure 4) and COVID-19 (Figure 6C) presentations. Thus, aside from any contribution to mechanistic understanding it might provide, the peak in late pollens could be exploited when contemplating an upcoming ILI season.

From an anthropologic perspective, the potential of mold spores and pollens to inhibit influenza-like epidemics/pandemics, including COVID-19, certainly has great relevance and significant consequence. Still, because in comparison to plants, fungi and even viruses, humans contribute only very little to the biomass on planet Earth [32], it seems likely some larger purpose is served by interplay between the three bioaerosols. It is tempting to speculate that any antiviral effect attributable to mold spores and/or pollens is intended to benefit primarily fungi and plants [33], i.e., the human benefit, albeit perhaps related mechanistically, is an epiphenomenon. As just one of many possibilities, mold spores and pollens might protect primarily arthropods, birds and bats, organisms intimately involved in dissemination of reproductive elements of both fungi and plants [34,35,36,37,38,39,40,41].

Separate and distinct from pollen count, mold spore count in Chicago correlates inversely with ILIs. Indeed, given their higher atmospheric concentration as well as the duration of their seasonal expression, mold spores seem more likely than pollens to be principals in any abatement of ILIs, including COVID-19. Because certain mold spores, e.g., *Aspergillus*, can propagate in man if left unattended by innate immune effectors, it also follows mold spores should be prioritized over pollens. Nonviral bioaerosols could abate viral activity by either direct or indirect means. By direct means, they might produce substances that limit viral propagation, or they might complex with viruses, limiting viral infectivity [42,43]. However, if direct antiviral activity is an attribute of the bioaerosols themselves, then one would not expect, a priori, significant disparity between individual susceptibilities to severe flu or COVID-19 [44,45]. As indirect means, others have proposed pollens stimulate the human immune system in such a way as to either potentiate endogenous antiviral activity or elicit a protective allergic response [3]. Against these proposals, asthma does not confer protection against either influenza or COVID-19 [46,47,48].

The similarity of the proposed mold spore dose dependencies for abatement of flu and COVID-19 suggests a shared receptor. Although much attention has been given to angiotensin-converting enzyme 2 (ACE-2) and its role in COVID-19 [12,13], there are compelling reasons to believe TLR4, which binds the SARS-CoV-2 spike protein with greater affinity than does ACE-2 [49], is also operative: 1) TLR4 is implicated in the inflammatory response triggered by sharply seasonal respiratory viruses [16,17,18], 2) TLR4 has a significant role in innate defense against multiple species of fungi [19,20] and polymorphisms in TLR4 are associated with invasive fungal disease [50,51], 3) COVID-19 prognosis correlates with radiographic involvement of alveolar spaces [21,22], the epithelial surfaces of which are poor in ACE-2 [52,53] but rich in TLR4 [23], (4) inflammation of the sort associated with acute lung injury is mediated by TLR4 [14,54,55,56,57,58,59,60,61,62], (5) age-dependent hyper-responsiveness of TLR4 [24], especially in the context of interactions with TLR5 [25,26], can account for the age-dependent severity of COVID-19 and (6) fibrino(gen) D-dimers are markedly elevated in persons with severe COVID-19 [28]. That TLR4 may be involved in the processing of bioaerosols is also expected on phylogenetic grounds: the receptor has been retained by some fish that breathe air but lost by those that do not [63], and the eponymous Toll receptor controls the antifungal response of *Drosophila* [64].

Given these, one can imagine the engagement of TLR4 by aerosols of all sorts and microscopic/submicroscopic sizes, including, but not limited to, viruses (diam 0.01–0.30 μm), mold spores (diam 1–50 μm) and pollens (diam 10–1000 μm), in a fashion analogous to the engagement of hook-and-loop adhesives, i.e., Velcro^®^. Instead of loops, however, spinous processes of the various aerosols engage TLR4 ‘hooks,’ effecting an innate immune response, the nature of which depends on the arrangement and density of the engagement. The large surface area of the extracellular domain of TLR4, 6000–8500 Å^2^ ensures accommodation of many such protuberances which, in turn, explains the broad specificity of the receptor [65]. In addition, just as hook-and-loop adhesives can be rendered nonfunctional/dysfunctional by nonspecific adherence of extraneous materials, so too might TLR4 hooks become saturated with one ligand to the exclusion of another. As for the role of fibrin(ogen) D-domains, their overexpression as endogenous ligands may represent an attempt by the innate immune system to purge/disengage TLR4 from pathogenic aerosols of all sorts for, perhaps, restorative purpose.

The data presented herein bring new appreciation and understanding to seasonality and suggest a remarkable interplay between bioaerosols that influence the health of man. Indeed, considering that humans have co-existed with plants, fungi and viruses for some time, it stands to reason that, over the course of evolution, the respiratory system of the former would have developed means to cope with the significant recurring, i.e., annual, inhalational exposure to reproductive elements of the latter. As the environment-facing interface of the respiratory tree, epithelial cells and their entourage of innate immune effectors seem ideally positioned to provide that coping mechanism. That being the case, nebulized materials that exploit competition either between the various bioaerosols or between the bioaerosols and endogenous TLR4 ligands, e.g., C-terminus of the fibrinogen γ-chain [66,67,68], might prove therapeutic. Finally, and notwithstanding allergic potential, the indoor cultivation—including mold-rich fertilization—of pollenating plants might be exploited to limit occurrence of sharply seasonal ILIs.

## Figures and Tables

**Table 1:** Bioaerosols of this study. The measured bioaerosols of this study are those listed here. They were collected and quantified as described in the text.

**Tree Pollens**
*Acer, Alnus, Betula, Carya, Cupressaceae, Cyperaceae, Fagus, Fraxinus, Juglans, Morus, Olea, Quercus, Pinaceae, Platanus, Populus, Salix, Tilia, Ulmus*
**Weed Pollens**
*Ambrosia, Artemisia, Asteraceae* (excluding *Ambrosia* and *Artemisia), Chenopodiaceae/Amaranthaceae, Liquidambar, Plantago, Rumex, Typha, Urticaceae*
**Grass Pollen**
*Gramineae/Poaceae*
**Mold Spores**
*Alternaria, Botrytis, Cercospora, Chaetomium, Cladosporium, Coprinus-type*, *Curvularia, Diatrypaceae, Dreshlera/Helminthosporium, Epicoccum, Fusarium, Ganaderma, Leptosphaeria-type, Nigrospora, Oidium/Erysiphe, Penicillium/Aspergillus, Periconia, Peronospora, Pithomyces, Pleospora, Polythrincium*, Rusts, Smuts/Myxomycetes, *Stemphylium, Torula*, undifferentiated Ascospores, undifferentiated Basidiospores, other fungi

**Table 2:** Kinetics of ILI presentations to emergency departments in Chicago. Both the rate of appearance and the rate of disappearance of ILI presentations to Chicago emergency departments are approximately first-order. Using the boundaries indicated, data were fit to first-order rate equations, which, for each year, were then solved for the parameters given in the table. See text for additional details and definitions of parameters. R^2^ is the coefficient of determination.

**Appearance**							
**Year**	**Date of Assigned Min.**	**Date of Assigned Max.**	**ILI_0_**, Presentations (95%)	**k**, d^−1^ (95%)	**Half-life**, d	**C**, Presentations (95%)	**R^2^**
2014	1/9/2015	3/30/2015	5 (3–7)	0.033 (0.028–0.042)	21	36 (33–39)	0.761
2015	10/27/2015	2/22/2016	1 (1–2)	0.035 (0.029–0.043)	20	40 (39–42)	0.699
2016	10/22/2016	2/19/2017	2 (1–6)	0.029 (0.022–0.038)	24	35 (28–40)	0.747
2017	10/9/2017	1/28/2018	19 (12–22)	0.021 (0.019–0.025)	34	0 (0–11)	0.882
2018	10/28/2018	3/20/2019	39 (36–42)	0.004 (0.003–0.005)	185	0 (0–0)	0.346
2019	11/11/2019	1/27/2020	49 (41–56)	0.016 (0.014–0.019)	43	0 (0–0)	0.673
2020	-	-	-	-	-	-	-
**Median**			**12**	**0.025**	**29**	**18**	
**Disappearance**							
**Year**	**Date of Assigned Max.**	**Date of Assigned Min.**	**ILI_0_**, Presentations (95%)	**k**, d^−1^ (95%)	**Half-life**, d	**C**, Presentations (95%)	**R^2^**
2014	-	-	-	-	-	-	-
2015	3/30/2015	8/4/2015	66 (61–72)	0.041 (0.034–0.049)	17	29 (26–31)	0.820
2016	2/22/2016	8/4/2016	113 (107–118)	0.031 (0.027–0.035)	22	18 (16–21)	0.899
2017	2/19/2017	9/3/2017	105 (99–110)	0.019 (0.017–0.022)	36	13 (9–16)	0.881
2018	1/28/2018	8/3/2018	155 (148–161)	0.028 (0.026–0.031)	24	20 (18–23)	0.935
2019	3/20/2019	8/20/2019	63 (59–68)	0.024 (0.020–0.029)	29	16 (13–19)	0.839
2020	3/18/2020	7/18/2020	145 (136–154)	0.026 (0.021–0.030)	27	10 (1–7)	0.897
**Median**			**109**	**0.027**	**26**	**17**	

**Table 3:** Contribution of pollens and mold spores to bioaerosol burden in Chicago. The pollens and mold spores of this study contribute variously to bioaerosol burden in Chicago. Pollens are listed in red. *Particle Count* is the number of pollen or mold spore particles counted over the period of the study, ~6 years. *% Total* is the percentage of all the particles counted.

Bioaerosol	Particle Count	% Total
*Cladosporium*	6,064,682	47.287
Undifferentiated Ascospores	3,598,475	28.058
Smuts/Myxomycetes	820,505	6.398
*Coprinus* *-type*	600,774	4.684
*Ganoderma*	368,568	2.874
*Alternaria*	224,356	1.749
*Cercospora*	208,950	1.629
*Diatrypaceae*	116,333	0.907
*Penicillium/Aspergillus*	109,272	0.852
*Epicoccum*	97,962	0.764
Undifferentiated Basidiospores	93,886	0.732
Rusts	93,107	0.726
*Dreshslera/Helminthosporium*	79,746	0.622
*Pithomyces*	45,102	0.352
*Leptosphaeria* *-type*	40,322	0.314
*Nigrospora*	36,701	0.286
*Torula*	32,652	0.255
*Morus*	28,019	0.218
*Periconia*	26,389	0.206
*Chaetomium*	14,408	0.112
*Curvularia*	14,166	0.11
*Oidium/Erysiphe*	12,012	0.094
*Stemphylium*	11,304	0.088
*Fusarium*	10,277	0.08
*Polythrincium*	9714	0.076
*Populus*	8034	0.063
*Quercus*	6792	0.053
*Acer*	6576	0.051
*Ambrosia*	5896	0.046
*Gramineae/Poaceae*	5399	0.042
*Peronospora*	4676	0.036
*Chenopodiaceae/Amaranthaceae *	4637	0.036
*Cupressaceae*	4395	0.034
*Betula*	4132	0.032
*Pinaceae*	2607	0.02
Unidentified Fungi	2366	0.018
*Urticaceae*	2337	0.018
*Plantago*	1584	0.012
*Pleospora*	1473	0.011
*Salix*	1284	0.01
*Artemisia*	1019	0.008
*Fraxinus*	823	0.006
*Ulmus*	822	0.006
Unidentified Pollen	699	0.005
*Rumex*	483	0.004
*Juglans*	386	0.003
*Alnus*	281	0.002
*Platanus*	231	0.002
*Tilia*	170	0.001
*Carya*	147	0.001
*Cyperaceae*	128	0.001
*Fagus*	120	0.001
*Liquidambar*	85	0.001
*Typha*	38	0
*Asteraceae (* excl. *Ambrosia* and *Artemisia)*	38	0
*Olea*	18	0
*Botrytis*	0	0

**Table 4:** Kinetics of mold spore expression in Chicago. Because the end date for counting bioaerosols in Chicago is fixed at approximately mid-October, the rate of disappearance of mold spores can only be approximated by the change in mold spore count over the interval of time indicated in the table. The rate of appearance of mold spores in Chicago is approximately first-order. Using the boundaries indicated, i.e., A and B, data were fit to first-order rate equations, which, for each year, were then solved for the parameters given in the table. See text for additional details. M_0_ is the calculated starting mold spore count, in particles/m^3^, at the beginning of an interval; k is the corresponding first-order rate constant, in d^−1^, and R^2^ is the coefficient of determination.

**Disappearance**							
**Year**	**Date of Assigned** **Maximum, A**		**Mold Spore Count on A**, particles/m^3^	**Date of Last** **Measurement, B**	**Mold Spore Count on B,** particles/m^3^	**Interval (A to B),** d	
2015	9/2/2015		76,403	10/16/2015	10,344	44	
2016	10/6/2016		101,028	10/21/2016	7203	15	
2017	9/25/2017		63,929	10/20/2017	10,519	25	
2018	9/5/2018		80,759	10/12/2018	22,466	37	
2019	9/20/2019		43,414	9/30/2019	12,920	10	
2020	9/4/2020		57,100	10/13/2020	5200	39	
**Median**			**70,166**		**10,432**	**31**	
**Appearance**							
**Year**	**Date of First Observed Appearance**	**Date of Assigned** **Maximum**	**Interval**, d	**M_0_**, particles/m^3^ (95%)	**k**, d^−1^ (95%)	**Half-life**, d	**R^2^**
2015	3/16/2015	9/2/2015	170	7350 (5427–9923)	0.008 (0.006–0.011)	84	0.211
2016	3/21/2016	10/6/2016	199	3759 (2788–5081)	0.012 (0.010–0.014)	59	0.478
2017	2/20/2017	9/25/2017	217	2682 (4418–7437)	0.011 (0.006–0.009)	65	0.395
2018	3/1/2018	9/5/2018	188	2256 (1489–3142)	0.015 (0.013–0.018)	45	0.572
2019	4/1/2019	9/24/2019	176	3137 (1399–3632)	0.013 (0.012–0.019)	52	0.634
2020	3/23/2020	9/4/2020	165	4044 (2820–5624)	0.012 (0.010–0.015)	56	0.479
**Median**			**188**	**3137**	**0.012**	**59**	

## Data Availability

The ILI and COVID-19 data of this study were obtained through portals of the Chicago Department of Public Health, the links for which are provided in the text.

## Supplementary Materials

The following are available online at https://www.mdpi.com/article/10.3390/pathogens10091204/s1, Table S1, Pollen Counts; Table S2, Mold Spore Counts; Table S3, ILI Presentations; Table S4, COVID-19 Presentations.

## References

[1] Lofgren E., Fefferman N.H., Naumov Y.N., Gorski J., Naumova E.N. (2007). Influenza Seasonality: Underlying Causes and Modeling Theories. J. Virol..

[2] Monto A.S., DeJonge P.M., Callear A.P., Bazzi L.A., Capriola S.B., Malosh R.E., Martin E.T., Petrie J.G. (2020). Coronavirus Occurrence and Transmission over 8 Years in the HIVE Cohort of Households in Michigan. J. Infect. Dis..

[3] Hoogeveen M.J., van Gorp E.C.M., Hoogeveen E.K. (2020). Pollen Explains Flu-like and COVID-19 Seasonality. medRxiv.

[4] Hoogeveen M.J. (2020). Pollen Likely Seasonal Factor in Inhibiting Flu-like Epidemics. A Dutch Study into the Inverse Relation between Pollen Counts, Hay Fever and Flu-like Incidence 2016–2019. Sci. Total Environ..

[5] Kanter U., Heller W., Durner J., Winkler J.B., Engel M., Behrendt H., Holzinger A., Braun P., Hauser M., Ferreira F. (2013). Molecular and Immunological Characterization of Ragweed (*Ambrosia artemisiifolia* L) Pollen after Exposure of the Plants to Elevated Ozone over a Whole Growing Season. PLoS ONE.

[6] Martinez A.T., Calvo M.A., Ramirez C. (1982). Scanning Electron Microscopy of *Penicillium* Conidia. Antonie Leeuwenhoek.

[7] Sannier J., Baker W.J., Anstett M.-C., Nadot S. (2009). A Comparative Analysis of Pollinator Type and Pollen Ornamentation in the Araceae and the Arecaceae, Two Unrelated Families of the Monocots. BMC Res. Notes.

[8] Silva D.M., Batista L.R., Rezende E.F., Fungaro M.H.P., Sartori D., Alves E. (2011). Identification of Fungi of the Genus *Aspergillus* Section *nigri* Using Polyphasic Taxonomy. Braz. J. Microbiol..

[9] Dijksterhuis J., van Egmond W., Yarwood A. (2020). From Colony to Rodlet. A Six Meter Long Portrait of the Xerophilic Fungus *Aspergillus restrictus* Decorates the Hall of the Westerdijk Institute. Fungal Biol..

[10] Paris S., Debeaupuis J.-P., Crameri R., Carey M., Charlès F., Prévost M.C., Schmitt C., Philippe B., Latgé J.P. (2003). Conidial Hydrophobins of *Aspergillus fumigatus*. Appl. Environ. Micro..

[11] Kurtzman C.P., Smiley M.J., Baker F.L. (1972). Scanning Electron Microscopy of Ascospores of *Schwanniomyces*. J. Bacteriol..

[12] Zhou P., Yang X.-L., Wang X.-G., Hu B., Zhang L., Zhang W., Si H.-R., Zhu Y., Li B., Huang C.-L. (2020). A Pneumonia Outbreak Associated with a New Coronavirus of Probably Bat Origin. Nature.

[13] Wang Q., Zhang Y., Wu L., Niu S., Song C., Zhang Z., Lu G., Qiao C., Hu Y., Yuen K.-Y. (2020). Structural and Functional Basis of SARS-CoV-2 Entry by Using Human ACE2. Cell.

[14] Sohn K.M., Lee S.-G., Kim H.J., Cheon S., Jeong H., Lee J., Kim I.S., Silwal P., Kim Y.J., Paik S. (2020). COVID-19 Patients Upregulate Toll-like Receptor 4-Mediated Inflammatory Signaling that Mimics Bacterial Sepsis. J. Korean Med. Sci..

[15] Aboudounya M.M., Heads R.J. (2021). COVID-19 and Toll-Like Receptor 4 (TLR4): SARS-CoV-2 May Bind and Activate TLR4 to Increase ACE2 Expression, Facilitating Entry and Causing Hyperinflammation. Med. Inflamm..

[16] Nhu Q.M., Shirey K., Teijaro J.R., Farber D., Netzel-Arnett S., Antalis T.M., Fasano A., Vogel S.N. (2010). Novel Signaling Interactions between Proteinase-Activated Receptor 2 and Toll-like Receptors In vitro and In vivo. Mucosal Immunol..

[17] Shirey K.A., Lai W., Brown L.J., Blanco J.C.G., Beadenkopf R., Wang Y., Vogel S.N., A Snyder G. (2020). Select Targeting of Intracellular Toll-Interleukin-1 Receptor Resistance Domains for Protection Against Influenza-Induced Disease. Innate Immun..

[18] Shirey K.a., Lai W., Patel M.C., Pletneva L.M., Pang C., Kurt-Jones E., Lipsky M., Roger T., Calandra T., Tracey K.J. (2016). Novel Strategies for Targeting Innate Immune Responses to Influenza. Mucosal Immunol..

[19] Hayes T., Rumore A., Howard B., He X., Luo M., Wuenschmann S., Chapman M., Kale S., Li L., Kita H. (2018). Innate Immunity Induced by the Major Allergen Alt a 1 from the Fungus Alternaria is Dependent upon Toll-like Receptors 2/4 in Human Lung Epithelial Cells. Front. Immunol..

[20] Roeder A., Kirschning C.J., Rupec R.A., Schaller M., Weindl G., Korting H.C. (2004). Toll-like Receptors as Key Mediators in Innate Antifungal Immunity. Med. Mycol..

[21] Francone M., Iafrate F., Masci G.M., Coco S., Cilia F., Manganaro L., Panebianco V., Andreoli C., Colaiacomo M.C., Zingaropoli M.A. (2020). Score in COVID-19 Patients: Correlation with Disease Severity and Short-term Prognosis. Eur. Radiol..

[22] Xiao J., Li X., Xie Y., Huang Z., Ding Y., Zhao S., Yang P., Du D., Liu B., Wang X. (2020). Maximum Chest CT Score is Associated with Progression to Severe Illness in Patients with COVID-19: A Retrospective Study from Wuhan, China. BMC Infect. Dis..

[23] Armstrong L., Medford A.R.L., Uppington K.M., Robertson J., Witherden I.R., Teresa T.D., Miller A.B. (2004). Expression of Functional Toll-like Receptor-2 and -4 on Alveolar Epithelial Cells. Am. J. Respir. Cell Mol. Biol..

[24] Hearps A.C., Martin G.E., Angelovich T., Cheng W.-J., Maisa A., Landay A.L., Jaworowski A., Crowe S.M. (2012). Aging is Associated with Chronic Immune Activation and Dysregulation of Monocyte Phenotype and Function. Aging Cell.

[25] Hussain S., Johnson C.G., Sciurba J., Meng X., Stober V.P., Liu C., Cyphert-Daly J.M., Bulek K., Qian W., Solis A. (2020). TLR5 Participates in the TLR4 Receptor Complex and Promotes MyD88-Dependent Signaling in Environmental Lung Injury. eLife.

[26] Qian F., Wang X., Zhang L., Chen S., Piecychna M., Allore H., Bockenstedt L., Malawista S., Bucala R., Shaw A.C. (2012). Age-Associated Elevation in TLR5 Leads to Increased Inflammatory Responses in the Elderly. Aging Cell.

[27] Zuliani-Alvarez L., Marzeda A., Deligne C., Schwenzer A., McCann F.E., Marsden B.D., Piccinini A.M., Midwood K.S. (2017). Mapping Tenascin-C Interaction with Toll-like Receptor 4 Reveals a New Subset of Endogenous Inflammatory Triggers. Nat. Comm..

[28] Hardy M., Michaux I., Lessire S., Douxfils J., Dogné J.-M., Bareille M., Horlait G., Bulpa P., Chapelle C., Laporte S. (2021). Prothrombotic Disturbances of Hemostasis of Patients with Severe COVID-19: A Prospective Longitudinal Observational Study. Thromb. Res..

[29] Hu W., Xie J., Chau H.W., Si B.C. (2015). Evaluation of Parameter Uncertainties in Nonlinear Regression Using Microsoft Excel Spreadsheet. Environ. Syst. Res..

[30] McKinnon G.H., Backhouse C.J., Kalantar A.H. (1984). Optimizing the use of the Kézdy-Mangelsdorf-Swinbourne Method for Analysis of Data Following A exp(-kt) + Z. Int. J. Chem. Kinet..

[31] Retzinger D.G., Retzinger A.C., Retzinger G.R. (2021). Estimate of Benefit Attributable to Wearing Masks in Chicago during the Early Days of the Pandemic. Med. Hypotheses.

[32] Bar-On Y.M., Phillips R., Milo R. (2018). The Biomass Distribution on Earth. Proc. Natl. Acad. Sci. USA.

[33] Noman A., Aqeel M., Qasim M., Haider I., Lou Y. (2020). Plant-Insect-Microbe Interaction: A Love Triangle between Enemies in Ecosystem. Sci. Total Environ..

[34] Biedermann P.H.W., Vega F.E. (2020). Ecology and Evolution of Insect—Fungus Mutualisms. Annu. Rev. Entomol..

[35] Kevan P.G., Baker H.G. (1983). Insects as Flower Visitors and Pollinators. Annu. Rev. Entomol..

[36] Rader R., Cunningham S.A., Howlett B.G., Inouye D.W. (2020). Non-Bee Insects as Visitors and Pollinators of Crops: Biology, Ecology, and Management. Annu. Rev. Entomol..

[37] da Silva L.P., Coutinho A.P., Heleno R.H., Tenreiro P.Q., Ramos J.A. (2016). Dispersal of Fungi Spores by Non-Specialized Flower-Visiting Birds. J. Avian Biol..

[38] Muchhala N., Thomson J.D. (2010). Fur versus Feathers: Pollen Delivery by Bats and Hummingbirds and Consequences for Pollen Production. Am. Nat..

[39] Diniz U.M., Lima S.A., Machado I.C.S. (2019). Short-Distance Pollen Dispersal by Bats in an Urban Setting: Monitoring the Movement of Vertebrate Pollinator through Fluorescent Dyes. Urban Ecosyst..

[40] Vanderwolf K.J., Malloch D., McAlpine D.F. (2016). Ectomycota Associated with Arthropods from Bat Hibernacula in Eastern Canada, with Particular Reference to *Pseudogymnoascus destructans*. Insects.

[41] Subudhi S., Rapin N., Misra V. (2019). Immune System Modulation and Viral Persistence in Bats: Understanding Viral Spillover. Viruses.

[42] Linnakoski R., Reshamwala D., Veteli P., Cortina-Escribano M., Vanhanen H., Marjomäki V. (2018). Antiviral Agents from Fungi: Diversity, Mechanisms and Potential Applications. Front. Microbiol..

[43] Pobiega K., Gniewosz M., Kraśniewska K. (2017). Antimicrobial and Antiviral Properties of Different Types of Propolis. Zesz. Probl. Postępów Nauk. Rol..

[44] Van Kerkhove M.D., Vandemaele K.A.H., Shinde V., Jaramillo-Gutierrez G., Koukounari A., Donnelly C.A., Carlino L.O., Owen R., Paterson B., Pelletier L. (2011). Risk Factors for Severe Outcomes Following 2009 Influenza A (H1N1) Infection: A Global Pooled Analysis. PLoS Med..

[45] Wang F., Cao J., Yu Y., Ding J., Eshak E.S., Liu K., Mubarik S., Shi F., Wen H., Zeng Z. (2020). Epidemiological Characteristics of Patients with Severe COVID-19 Infection in Wuhan, China: Evidence from a Retrospective Observational Study. Int. J. Epidemiol..

[46] Walker T.A., Waite B., Thompson M.G., McArthur C., Wong C., Baker M.G., Wood T., Haubrock J., Roberts S., Gross D.K. (2020). Risk of Severe Influenza among Adults with Chronic Medical Conditions. J. Infect. Dis..

[47] Chhiba K.D., Patel G.B., Vu T.H.T., Chen M.M., Guo A., Kudlaty E., Mai Q., Yeh C., Muhammad L.N., Harris K.E. (2020). Prevalence and Characterization of Asthma in Hospitalized and Nonhospitalized Patients with COVID-19. J. Allergy Clin. Immunol..

[48] Javanmardi F., Keshavarzi A., Akbari A., Emami A., Pirbonyeh N. (2020). Prevalence of Underlying Diseases in Died Cases of COVID-19: A Systematic Review and Meta-analysis. PLoS ONE.

[49] Choudhury A., Mukherjee S. (2020). In silico Studies on the Comparative Characterization of the Interactions of SARS-CoV-2 Spike Glycoprotein with ACE-2 Receptor Homologs and Human TLRs. J. Med. Virol..

[50] Koldehoff M., Beelen D.W., Elmaagacli A.H. (2013). Increased Susceptibility for Aspergillosis and Post-Transplant Immune Deficiency in Patient with Gene Variants of TLR4 after Stem Cell Transplantation. Transpl. Infect. Dis..

[51] Carvalho A., Pasqualotto A.C., Pitzurra L., Romani L., Denning D.W., Rodrigues F. (2008). Polymorphisms in Toll-like Receptor Genes and Susceptibility to Pulmonary Aspergillosis. J. Infect. Dis..

[52] Hikmet F., Méar L., Edvinsson Å., Micke P., Uhlén M., Lindskog C. (2020). The Protein Expression Profile of ACE2 in Human Tissues. Mol. Syst. Biol..

[53] Lee I.T., Nakayama T., Wu C.-T., Goltsev Y., Jiang S., Gall P.A., Liao C.-K., Shih L.-C., Schürch C.M., McIlwain D.R. (2020). ACE2 Localizes to the Respiratory Cilia and is Not Increased by ACE Inhibitors or ARBs. Nat. Commun..

[54] Lee N., Wong C.K., Hui D., Lee S.K.W., Wong R.Y.K., Ngai K.L.K., Chan M.C.-W., Chu Y.J., Ho A.W.Y., Lui C.Y.G. (2013). Role of Human Toll-like Receptors in Naturally Occurring Influenza A Infections. Influenza Other Respir. Viruses.

[55] Marchant D., Singhera G.K., Utokaparch S., Hackett T.L., Boyd J.H., Luo Z., Si X., Dorscheid D.R., McManus B.M., Hegele R.G. (2010). Toll-like Receptor 4-Mediated Activation of p38 Mitogen-Activated Protein Kinase is a Determinant of Respiratory Virus Entry and Tropism. J. Virol..

[56] Shoenfelt J., Mitkus R.J., Zeisler R., Spatz R.O., Powell J., Fenton M.J., Squibb K.A., Medvedev A.E. (2009). Involvement of TLR2 and TLR4 in Inflammatory Immune Responses Induced by Fine and Coarse Ambient Air Particulate Matter. J. Leucoc. Biol..

[57] Figueiredo R.T., Carneiro L.A.M., Bozza M.T. (2011). Fungal Surface and Innate Immune Recognition of Filamentous Fungi. Front. Microbiol..

[58] Bourgeois C., Kuchler K. (2012). Fungal Pathogens—a Sweet and Sour Treat for Toll-like Receptors. Front. Cell Infect. Microbiol..

[59] Hosoki K., Boldogh I., Sur S. (2015). Innate Response to Pollen Allergens. Curr. Opin. Allergy Clin. Immunol..

[60] Dahl Å. (2018). Pollen Lipids can Play a Role in Allergic Airway Inflammation. Front. Immunol..

[61] Imai Y., Kuba K., Neely G., Yaghubian-Malhami R., Perkmann T., Van Loo G., Ermolaeva M., Veldhuizen R., Leung Y.C., Wang H. (2008). Identification of Oxidative Stress and Toll-like Receptor 4 Signaling as a Key Pathway of Acute Lung Injury. Cell.

[62] Hu R., Xu H., Jiang H., Zhang Y., Sun Y. (2013). The Role of TLR4 in the Pathogenesis of Indirect Acute Lung Injury. Front. Biosci..

[63] Palti Y. (2011). Toll-like Receptors in Bony Fish: From Genomics to Function. Develop. Comp. Immunol..

[64] Lemaitre B., Nicolas E., Michaut L., Reichhart J.-M., Hoffman J.A. (1996). The Dorsoventral Regulatory Gene Cassette spätzle/Toll/cactus Controls the Potent Antifugal Response in Drosophila Adults. Cell.

[65] Bell J., Mullen G.E., Leifer C.A., Mazzoni A., Davies D.R., Segal D.M. (2003). Leucine-rich Repeats and Pathogen Recognition in Toll-like Receptors. Trends Immunol..

[66] Yee V.C., Pratt K.P., Cote H.C., Le Trong I., Chung D.W., Davie E.W., Stenkamp R.E., Teller D.C. (1997). Crystal structure of a 30 kDa C-Terminal Fragment from the γ Chain of Human Fibrinogen. Structure.

[67] Doolittle R.F., McNamara K., Lin K. (2012). Correlating Structure and Function during the Evolution of Fibrinogen-Related Domains. Prot. Sci..

[68] Zuliani-Alvarez L., Midwood K.S. (2015). Fibrinogen-Related Proteins in Tissue Repair: How a Unique Domain with a Common Structure Controls Diverse Aspects of Wound Healing. Adv. Wound Care.

